# Supplementary material for: The epidemiology of HIV population viral load in twelve sub-Saharan African countries
Source: PLoS One. 2023 Jun 26;18(6):e0275560. doi: 10.1371/journal.pone.0275560 (PMC10292693; doi:10.1371/journal.pone.0275560)
Supplement: S1 Table — (DOCX) [file pone.0275560.s001.docx]

**S1 Table**: Adult population, PLHIV, and number unsuppressed at time of survey and adjustments in creation of raster-based population counts

|  | | **PHIA sample-based population estimates (weighted)** | | | | **National population totals using WorldPop [**28**] pixel level population estimates (2020)** | | | | | | |  |  |
| --- | --- | --- | --- | --- | --- | --- | --- | --- | --- | --- | --- | --- | --- | --- |
| Country | | Adult population size | | No. PLHIV | No. unsuppressed PLHIV |  | | | | | |  |  |  |
|  |  |  |  |  |  | WorldPop total population ∑Total(pixel) | Adult population adjustment multiplier | WorldPop total adult PLHIV ∑PLHIV(pixel) | PLHIV adjustment multiplier | Worldpop total unsuppressed adult PLHIV ∑Viremic(pixel) | Unsuppressed population adjustment multiplier |  |  |  |
|  | | (a) | | (b) | (c) | (d) | (e)=(a)/(d) | (f) | (g)=(b)/(f) | (h) | (i)=(c)/(h) |  |  |  |
| Cameroon | | 13,542,765 | | 499,863 | 276,556 | 26,498,058 | 0.5111 | 494,185 | 1.0115 | 278,273 | 0.9938 |  |  |  |
| Cote d'Ivoire | | | 13,690,917 | 381,907 | 227,401 | 26,364,963 | 0.5193 | 337,457 | 1.1317 | 196,315 | 1.1584 |  |  |  |
| Kenya | | 26,670,803 | | 1,303,268 | 370,422 | 53,366,821 | 0.4998 | 1,331,792 | 0.9786 | 369,891 | 1.0014 |  |  |  |
| Lesotho | | 1,196,696 | | 305,853 | 98,979 | 2,142,223 | 0.5586 | 313,939 | 0.9742 | 96,776 | 1.0228 |  |  |  |
| Malawi | | 8,529,257 | | 901,341 | 284,271 | 19,127,635 | 0.4459 | 1,003,246 | 0.8984 | 295,440 | 0.9622 |  |  |  |
| Namibia | | 1,402,000 | | 176,329 | 39,857 | 2,533,083 | 0.5535 | 202,882 | 0.8691 | 43,380 | 0.9188 |  |  |  |
| Rwanda | | 7,012,336 | | 210,199 | 50,416 | 12,952,096 | 0.5414 | 212,691 | 0.9883 | 50,493 | 0.9985 |  |  |  |
| Eswatini | | 689,787 | | 192,387 | 52,249 | 1,154,797 | 0.5973 | 195,268 | 0.9852 | 49,375 | 1.0582 |  |  |  |
| Tanzania | | 29,790,930 | | 1,494,555 | 716,490 | 59,686,630 | 0.4991 | 1,591,994 | 0.9388 | 747,341 | 0.9587 |  |  |  |
| Uganda | | 19,132,600 | | 1,195,299 | 482,647 | 45,707,935 | 0.4186 | 1,235,257 | 0.9677 | 482,888 | 0.9995 |  |  |  |
| Zambia | | 8,005,534 | | 960,665 | 383,037 | 18,383,938 | 0.4355 | 1,048,313 | 0.9164 | 398,553 | 0.9611 |  |  |  |
| Zimbabwe | | 8,174,858 | | 1,152,520 | 464,792 | 14,862,923 | 0.5500 | 1,277,823 | 0.9019 | 481,045 | 0.9662 |  |  |  |
| Total | | 150,523,667 | | 9,158,199 | 3,561,832 |  |  |  |  |  |  |  |  |  |
| Notes: | Columns (a) through (c) are weighted PHIA survey estimates and reflect estimated number individuals and percent prevalences at the time of survey | | | | | | | | | | | |  |  |
|  | (d) sum of the number of people per pixel from WorldPop raster maps (based on 2019 United Nations population projections for 2020) | | | | | | | | | | | |  |  |
|  | (e) adjustment applied to WorldPop raster maps to obtain adults population per pixel (circa mid-2020), computed as (a)/(d) | | | | | | | | | | | | |  |
|  | (f) sum of number of adult PLHIV obtained from multiplying HIV prevalence by the adult population per pixel (circa mid-2020) | | | | | | | | | | | | |  |
|  | (g) adjustment applied to adult PLHIV raster (based on 2020 projection) to obtain expected counts at time of the survey, computed as (b)/(f) | | | | | | | | | | | |  |  |
|  | (h) sum of number of adult unsuppressed PLHIV obtained from multiplying unsuppressed prevalence by the adult population per pixel (circa mid-2020) | | | | | | | | | | | |  |  |
|  | (i) adjustment applied to unsuppressed PLHIV adult raster (based on 2020 projection) to obtain expected counts at time of the survey, computed as (c)/(h)  PLHIV: People living with HIV; PHIA: Population-based HIV Impact Assessment. | | | | | | | | | | | |  |  |
