# Supplementary material for: The epidemiology of HIV population viral load in twelve sub-Saharan African countries
Source: PLoS One. 2023 Jun 26;18(6):e0275560. doi: 10.1371/journal.pone.0275560 (PMC10292693; doi:10.1371/journal.pone.0275560)
Supplement: S1 File — (DOCX) [file pone.0275560.s004.docx]

**Supporting information**: US and local IRB approval documentation.

| **Country** | **CDC Prevention IRB** | | **CUMC IRB** | | **Westat IRB** | | **Local and Additional IRB Institutions** | | |
| --- | --- | --- | --- | --- | --- | --- | --- | --- | --- |
|  | **Protocol Number** | **Approval Date** | **Protocol Number** | **Approval Date** | **Protocol Number** | **Approval Date** | **IRB Name(s)** | **Protocol Number(s)** | **Approval Date(s)** |
| Cameroon | 6950 | 26-Jan-17 | IRB-AAAR2282 | 8-Mar-17 | 6317 | 19-May-15 | Comite National D'Ethique De La Recherche Pour La Sante Humaine (CNERSH) | 2017/02/868/CE/CNERSH/SP | 16-Feb-17 |
| Cote d'Ivoire | 7019 | 11-Jul-17 | IRB-AAAR4152 | 6-Jul-17 | 6317 | 19-May-15 | Comite National D'Ethique De La Recherche (CNER) | 064-18/MSHP/CNER-km | 6-Jun-17 |
| Eswatini | 6846 | 14-Apr-16 | IRB-AAAQ8889 | 6-Jul-16 | 6420 | 22-Mar-16 | Swaziland Ethics Committee (SEC) | MH/599C/FWA 000 15267/ IRB 000 9688 | 9-Jun-16 |
| Kenya | 7094 | 30-Mar-18 | IRB-AAAR7792 | 30-Mar-18 | 6317 | 19-May-15 | Kenya Medical Research Institute (KEMRI) | KEMRI/RES/7/3/1 | 6-Dec-17 |
| Lesotho | 6883 | 1-Nov-16 | IRB-AAAQ8537 | 15-Jun-16 | 6317 | 19-May-15 | Ministry of Health Research and Ethics Committee | ID60-2016 | 8-Apr-16 |
| Malawi | 6692 | 4-Mar-15 | IRB-AAAO9051 | 15-Apr-15 | 6264 | 31-Oct-14 | National Health Sciences Research Committee (NHSRC) | NHSRC # 1361 | 30-Apr-15 |
| Namibia | 6943 | 17-Apr-17 | IRB-AAAR2051 | 22-Mar-17 | 6317 | 19-May-15 | Ministry of Health and Social Sciences (MOHSS); UCSF Human Research Protection Program IRB | 17/3/3 AN; 17-21642 | 21-Apr-17; 10-Apr-17 |
| Rwanda | 7157 | 24-Aug-18 | IRB-AAAR8357 | 30-Aug-18 | 6317 | 19-May-15 | Rwanda National Ethics Committee (RNEC) | 103/RNEC/2018 | 13-Apr-18 |
| Tanzania | 6880 | 27-Jul-16 | IRB-AAAQ7860 | 28-Jul-16 | 6317 | 19-May-15 | National institute of Medical Research (NIMR); Zanzibar Research Institute (ZAMREC) | NIMR/HQ/R8a; ZAMREC/0001/February/2015 | 24-Feb-16; 19-Feb-16 |
| Uganda | 6830 | 9-Mar-16 | IRB-AAAQ8408 | 21-Jun-16 | 6692 | 19-May-15 | Uganda Virus Institute (UVRI); Uganda National Council for Science and Technology (UNCST) | GC/127/15/07/43; SS 4085 | 14-Jan-16; 27-Jun-16 |
| Zambia | 6760 | 26-Aug-15 | IRB-AAAQ0753 | 4-Oct-15 | 6317 | 19-May-15 | Tropical Diseases Research Center Ethics Committee (TDRC); The National Health Research Authority (NHRA) | TRC/C4/18/2015; MH/101/23/10-1 | 18-Dec-15; 29-Feb-16 |
| Zimbabwe | 6702 | 1-Jun-15 | IRB-AAAP0807 | 27-May-15 | 6264 | 31-Oct-14 | Medical Research Council of Zimbabwe (MRCZ) | MRCZ/A/1914 | 5-Sep-15 |
| CDC: Centers for Disease Control and Prevention, Atlanta, USA; CUMC: Columbia University Medical Center (New York, New York); IRB: Institutional review board; Approval Date: refers to the initial date the survey protocol was approved. Note: For Namibia, an additional US-based IRB institution included UCSF (University San Francisco California). | | | | | | | | | |
