# Supplementary material for: The epidemiology of HIV population viral load in twelve sub-Saharan African countries
Source: PLoS One. 2023 Jun 26;18(6):e0275560. doi: 10.1371/journal.pone.0275560 (PMC10292693; doi:10.1371/journal.pone.0275560)
Supplement: S2 File — (DOCX) [file pone.0275560.s005.docx]

Inclusivity in global research

PLOS’ policy on inclusivity in global research aims to improve transparency in the reporting of research performed outside of researchers’ own country or community and ensures that PLOS publications reporting global research adhere to high standards for research ethics and authorship. Authors of relevant research articles may be asked to complete the questionnaire below, which outlines ethical, cultural, and scientific considerations specific to inclusivity in global research. This questionnaire may be requested when researchers have travelled to a different country to conduct research, if research uses samples collected in another country, research with Indigenous populations or their lands, or if research is on cultural artefacts. Researchers travelling to another country solely to use laboratory equipment will not normally be required to complete the questionnaire. However, the questionnaire can be requested at the journal’s discretion for any submission – if you have been requested to complete this questionnaire by the PLOS journal you submitted to, please do so.

Please complete the questionnaire below and include this as a Supporting Information file with your manuscript. Note that if your paper is accepted for publication, this checklist will be published with your article in the supporting information files. Please ensure that you reference the checklist in the main body of your manuscript. We suggest adding a subsection ‘Inclusivity in global research’ to your Methods section and adding the following sentence: “Additional information regarding the ethical, cultural, and scientific considerations specific to inclusivity in global research is included in the Supporting Information (SX Checklist)”

The questions have been designed to be applicable to a wide range of study types, and there are subsections for both human subjects research and non-human subjects research. If any of the questions are not relevant to your research please mark them as “N/A” as appropriate.

**Ethical considerations, permits and authorship**

*This section is applicable to all research types.*

Provide details as to who granted permissions and/or consent for the study to take place in the Methods section of your manuscript. This should include the names of **all** ethics boards, governmental organizations, community leaders or other bodies that provided approval for the study. If individuals provided approval refer to these people by their role or title but do not list their name(s).

Reported on page number:

If there were any deviations from the study protocol after approval was obtained please provide details of these changes in the Methods section of your manuscript.
Did this study involve local collaborators that are residents of the country where the research was conducted or members of the community studied? If you do not have any authors from said communities, please provide an explanation for this below.

Reported on page number:

Yes, each country’s national survey was conducted in collaboration with the ministry of health and national statistics office and survey protocols included PIs and other investigators from each organization. The adaptation of PHIA materials in each country was done locally via technical working groups comprised of local subject matter experts from government and local HIV organizations, including those representing the HIV-positive community, and other local civil society organizations. Survey implementation and management staff were hired locally in the country the survey was conducted in. Each survey had a local scientific publication committee that ensured equitable access to data prior to public release and opportunities for publication were equitably distributed among investigating institutions and each country had representation on a multicounty publication committee for the same purposes. Though this data was already public, these committees were involved during the development of the manuscript to offer co-authorship opportunities to individuals from all 12 countries.

Everyone listed as an author should meet PLOS’ criteria for authorship and all individuals who meet these criteria should be included in the author byline, rather than the acknowledgements. Authorship criteria is based on the International Committee of Medical Journal Editors (ICMJE) Uniform Requirements for Manuscripts Submitted to Biomedical Journals - for further information please see here: <https://journals.plos.org/plosone/s/authorship>.

**Human subjects research (e.g. health research, medical research, cross-cultural psychology)**

Did you obtain written informed consent from a representative of the local community or region before the research took place? How did you establish who speaks for the community? Details of written informed consent obtained from study participants should be reported separately in the Methods section of your manuscript.

Prior to working in a given administrative area (e.g. district), approval for data collection was obtained through the appropriate government structures. Prior to starting field data collection, survey teams, comprised of local residents, visited selected survey areas as part of the survey’s community mobilization activities. During these visits, meetings were held with communities, community representatives, and local leaders and information about the upcoming survey was disseminated.

How did members of the local community provide input on the aims of the research investigation, its methodology, and its anticipated outcome(s)?

The adaptation of PHIA materials in each country was done locally via technical working groups made of up local subject matter experts from government and local HIV organizations, including those representing the HIV-positive community, and other local civil society organizations. The local (subnational) community did not provide direct input into survey protocol development – these communities were selected for the sample after the protocol was developed. At the national level, survey protocols were developed by CDC, ICAP, Westat, Ministries of Health, and other local institutions. Of note, these surveys are observational in nature.

When engaging with the local community, how did you ensure that the informed consent documents and other materials could be understood by local stakeholders?

In all countries, informed consent documents, community-facing materials (e.g. posters), and participant-facing materials (e.g. brochures) were translated into the appropriate local languages. All field data collection, including administration of informed consent, was conducted by local survey staff, fluent in the language.

Will the findings of the research be made available in an understandable format to stakeholders in the community where the study was conducted (e.g. via a presentation, summary report, copies of publications, etc.)? Please provide details of how this will be achieved.

Survey summary and full survey reports – the primary dissemination products - for all 12 surveys have been published and disseminated already, including at local events in all 12 countries. Copies of these reports and summaries are available to all online. This manuscript is based on a secondary analysis of the publicly available data.

**Non-human subjects research using specimens/ animals collected as part of the study, or those housed in archival collections. Examples include archaeology, paleontology, botany and zoology.**

Did the permission you obtained from a local authority to perform the study include an agreement on access to outputs and benefit sharing? This may include procedures to enable fair distribution of the benefits and resources arising from the research performed. Please include any details of Prior Informed Consent and Benefit Sharing Agreements obtained. These may be required by field-specific regulations, for example the Convention on Biological Diversity (CBD) and the associated Nagoya Protocol.

NA

If the material used in your study was imported, please A) provide the year it was imported and B) indicate whether permits were obtained to import/export the materials used, C) provide details of any permits obtained. If this information is not available, please indicate this.

NA

If you used archival specimens, please state how the material used in your study was acquired by the institute it is held in and provide details of any permits obtained for the original excavations/ sample collection. If this information is not available, please indicate this.

NA

How was the potential cultural significance of the materials collected in your study to local communities considered in your research design? Were Indigenous peoples and/or local researchers and institutions involved with archaeological excavations / collection of specimens? If so, please provide a description of their involvement.

NA

If your manuscript includes photographs of human remains please indicate whether authors obtained permission from descendants or affiliated cultural communities to do so.

NA
