# Supplementary material for: The epidemiology of HIV population viral load in twelve sub-Saharan African countries
Source: PLoS One. 2023 Jun 26;18(6):e0275560. doi: 10.1371/journal.pone.0275560 (PMC10292693; doi:10.1371/journal.pone.0275560)
Supplement: Attachment — Submitted filename: Rebuttal Letter - PONE-D-22-26006 - Jan 2023.docx [file pone.0275560.s006.docx]

The authors greatly appreciate the reviewers’ time and thoughtful comments. Please find our point-to-point responses in purple below. Thank you.

5. Review Comments to the Author

Please use the space provided to explain your answers to the questions above. You may also include additional comments for the author, including concerns about dual publication, research ethics, or publication ethics. (Please upload your review as an attachment if it exceeds 20,000 characters)

Reviewer #1: The manuscript by Dr Hladik and colleagues reports on the epidemiology of HIV population viral load in twelve sub-Saharan African countries. The authors used data generated in the framework of Population-based HIV Impact Assessments (PHIAs) conducted between 2015 and 2019 to perform their analyses. These were well performed, with appropriate methods. The manuscript, although very long and dense, is well written. The findings presented are important at both country and international levels. The authors are thus commended for their endeavor.

The work deserves some comments.

Major

1. The major comment I have is that the work was performed in 12 sub-Saharan African countries but the first 7 and the last two authors are from outside Africa. This is not normal and the authors should change that before any other further consideration.

Response: We appreciate this comment and would like to offer clarification: All data sets used for this manuscript are in the public domain and this work represents a secondary analysis using data from 12 surveys. The idea, conceptualization, analysis, and manuscript drafting was led by the first three authors. The last two authors are affiliated with CDC and Columbia University headquarters who had leading roles in survey design or supported many of these surveys. In each country, we solicited authorship from MoH staff, as well as local CDC and Columbia/ICAP staff. All authors were survey investigators and or reviewed the manuscript.

Minor

1. On page 8, line 155, it is written that HIV-2 data were excluded from the analysis. Can the authors indicate how many of such cases were recorded, presumably all from Côte d’Ivoire?	Response: 59 respondents in Côte d’Ivoire tested HIV-2-positive; of these, 40 also tested HIV-1-positive. For the purpose of our analysis, HIV status and prevalence was based on HIV-1 testing results.

2. On page 4, line 68in the introduction, it is written that VLS eliminates the risk of HIV transmission. The world eliminates is too strong. Maybe “reduces” or “minimizes” as written line 71 of the same page is more appropriate.
Response: Thank you, we changed the writing as suggested.

3. One factor that was not considered in the analysis in the 12 countries is HIV-1 diversity as explaining factor of observed differences. Between countries of Wes-Central Africa, those of Easter Africa and finally those of Southern Africa, different HIV-1 subtypes are circulating, which slightly differ in their fitness, susceptibility to ART and transmissibility. The authors should mention in the discussion these possibilities also.
Response: Agreed. We altered the sentence on p22 (line 113) to say: *Further, we could not consider the contribution of HIV subtypes or HIV disease stage (primary, latent, late stage) to HIV transmission,…*

4. In the discussion section, the authors mentioned male circumcision (page 26-27). This parameter is not the same in West and Central Africa, where most of the males are circumcised, and in the Southern Africa. I think giving at least the proportion of circumcised males in different countries remain an interesting information to share.
Response: We added the sentence “*Data from the same surveys suggest that the self-reported prevalence of medical male circumcision varied from 9.8% (Malawi) to 58.1% (Cameroon).*”

5. Finally, I find the discussion quite long.

Reviewer #2: I make this review in my personal capacity and not as a staff of UNAIDS.

The authors have worked hard, demonstrating a strong grasp of the topic. However, the paper is not ready for publication.
With major revisions this paper may make an important contribution to the body of knowledge on viral load metrics.

The questions being investigated and why they are being investigated need clear explanation, and some of the main literature a closer read, to determine if the approaches undertaken are valid.

For example, where does 0 start for the Lorenzo curve. Is it at less than 1K HIV viral load, since undetectable does not contribute to the total viral load? Has the viral load measurement been lognormalised, or scaled in way that is easier to plot?

Response: First we sorted all observations for HIV+ persons by the weighted number of viral copies.  Then, 13 population groups were formed by calculating the cumulative percent of the population, counting from the observation with the lowest VL value to the highest value. The 13 groups were formed by setting cut points of the cumulative percent of population at 10, 20, 30, 40, 50, 60, 70, 80, 90, 95, 98, 99, and 100 percent).  Then the weighted total VL within each of the 13 groups was divided by total VL across all 13 groups to derive the weighted percent VL for each group. These percentages were then cumulated and are plotted as the y-axis of the Lorenz curve.  If the VL for an individual was below the threshold for detection, they were assigned a value of 1.0. We added similar text to the manuscript (“*Undetectable VL values were assigned a VL value of 1 for the construction of the Lorenz curves*.”)

As an example, in Cameroon for the lowest 10 percent of the population the total VL was 49,874 copies, but the estimated grand total in the entire population of PLHIV was 78,592,554,644 copies. The percent of all copies in the lowest 10 percent of the PLHIV population is .00001, which appears to be zero on the Lorenz curve.

We believe a log scale here would not be useful since what we are plotting are cumulative percentages (not VL counts).

Further, it is not clear if a viral load of 3000 is better than a viral load of 4000 or 5000 in terms of HIV transmission dynamics. For this reason, the lorenz curve and Ginie co-efficient may not be an appropriate method to measure inequality in viral load suppression because HIV viral load does not have the same properties as income or wealth. Maybe remove the lorenz curve analysis.

Response: Regarding the Lorenz curves (and Gini coefficients), the inequality displayed through this graph and statistic is primarily a way to display the country-level effects of ART programs leading to this desirable state of inequality, and, we believe, to show how the remaining unsuppressed VL is concentrated in a relatively small proportion of PLHIV. While the difference between a VL of 3000 and 5000 may be small or negligible in terms of HIV transmission risk, the literature suggests this risk difference is appreciable overall and on a natural log scale, i.e., higher/very high VL values are one determinant for HIV transmission risk (along with other, eg behavioral factors).

And also see which other analysis you may remove to improve the clarity of the paper. The metrics although few are each complex and not easy to follow. Focusing on one of two metrics may improve the usefulness of the analysis.

Response: One of the purposes of this manuscript is to demonstrate the many ways of examining VL. While removing the Lorenz curves and perhaps other metrics would shorten the paper we feel the shorter manuscript would also lose in value that way.

Table 2 shows promise if its better explained and standardized. For example, the standard errors are only included in the HIV transmission viremia and not in others. And also, the standard errors are large and there is no discussion of these standard errors, if they robust standard errors. Maybe remove the standard errors.
Response: Agreed; we removed the standard errors.

Overall, the tables, graphs and maps are well done. They would be clear if the axis are labelled and each table or figure briefly described before presenting the results. If possible, put the description of each table or figure on the same page to help the reviewer follow what each figure or table is conveying, depending on what the journal requires.
Response: Sorry for this; we followed the manuscript submission guidelines and believe this layout is what PLoS One expects.

Figure 1 is not clear what is being plotted, 20 is less than 1k in the Table so the header of second column should be 1K. Similarly figure 20 is less than 100.
Response: We edited the caption for Fig 1 and the x-axis VL category labels in Fig. They now read, e.g., *20 to 1K, 1000 to <10K,* etc.

For the maps indicate the units of measurements as you did for the population graphs e.g. 100s or 1,000S. Also see if you want to present both prevalence and viremia because the information appear not too different between the two, after addressing the earlier comments on calculating population viremia.
Response: Based on previous, internal reviewer comments for this paper, we included all three versions of the country maps, i.e., HIV prev, viremia prev and No. viremic PLHIV. The unit of measurement is roughly indicated in the scaling legend next to each map (#viremic); the coloring indicating the number of viremic PLHIV (per pixel) differs for each country, reflecting widely differing total population size and # PLHIV across countries.

Please see the following points that may help improve the paper.
dited
Include the data set from Ethiopia since it is available. If not indicate why you excluded the data set from Ethiopia. Because it was conducted only in urban areas may not be sufficient reason to exclude it.
Response: We added text (page 6, subsection Study Population) to explain why we excluded Ethiopia, which is, as you suspected, because only urban Ethiopia was sampled, hence impeding 1) national VL metrics, 2) estimating HIV transmission ratios, 3) examining the relationship between viremia and HIV incidence, and 4) the creation of a national map displaying the burden of HIV and viremia.

Change female to women, male to men after the first mention, through out the paper.
Response: Where the sentence phrasing was fitting, we made a number of these changes as suggested.

Include the names of the country of the sample size. I did not see which country has a sample size of 30,637 indicated in the abstract. Please check.
Response: Country sample sizes are shown on page 6, subsection Study Population. Rwanda had a sample size of 30,637.

"The number of viremic women outnumbered the viremic men in all countries" Is an important finding. Please present this result also in proportions and devote maybe a paragraph or so discussing the results and its policy implications.
Response: As suggested, we added a row in table 1 to display the proportion female among unsuppressed PLHIV and reference the overall proportion of unsuppressed PLHIV among females (median: 59%). In the manuscript’s Discussion section, we already cover this observation: “Although females generally achieve higher proportions of VLS compared to males in all PHIA countries, examining the absolute numbers of unsuppressed PLHIV suggests that in all countries viremic females still outnumber viremic males, due to the higher HIV prevalence observed among adult females. This finding may serve as a reminder that while more progress towards VLS among men is warranted, most of the absolute viremic HIV burden in SSA continues to be shouldered by females.”

On page 4, please read carefully the new UNAIDS Strategy and paraphrase from this strategy information on line 66 - 68 to something like "focuses on eliminating inequalities in access to HIV services...." It may not be correct to present the strategy as focusing on treatment as prevention.
Response: Thank you for pointing this out. We altered the sentence as suggested.

Also, on line 71 include to be met by 2025 next to the 95-95-95.
Response: Thank you, edited as suggested.

Line 77 "... correlated with (add) HIGHER..."
Response: Altered as suggested.

Line 80, please rephrase the sentence after the sources, becuase dichotomizing VL is useful as an analytic variable. It helps policy makers to track viral load suppression
Response: We altered the sentence to say “Further, while dichotomizing VL into suppressed and unsuppressed has utility for programming and policy, the categorization also leads to information loss as the level of VL is strongly correlated with HIV disease progression and transmission probability.”

Line 155 please explain why this was excluded.
Response: We altered the text to say “(HIV-2 data were excluded from analysis due to the very different relationship between VL, disease progression, and transmission risk)”

On line 161 include the laboratory confirmation of results.
Response: On page 7, we write “All HIV-positive specimens underwent confirmatory testing using the Geenius HIV 1/2 Supplemental Assay (Bio-Rad, Hercules, CA, USA) or PCR testing. Additional HIV quality control testing varied by country.”

Line 171. It may not be correct to multiply the HIV prevalence with the proportion of people who were unsuppressed. With this approach countries such as Eswatini with higher HIV prevalence will have lower population HIV viral load, for example, for the same proportion of people with unsuppressed viral loads.
Response: Your example is correct and our metric is correct as well. Population viremia is sensitive to both HIV prevalence and (inversely) VLS. As such, countries with differing HIV prevalence and VLS suppression may display similar population viremia. Example: Country A – HIV prevalence 10%, VLS 50% (proportion unsuppressed: 50%); country B – HIV prevalence 15%, VLS 66.7% (proportion unsuppressed: 33.3%), both countries will have a population viremia of 5%.

Kindly add the total of values unsuppressed viral load to find the total national value of HIV viral load that is not suppressed.
Response: Table 1 displays each country’s total VL, both on a logarithmic and non-log scale. These sum values include both unsuppressed and suppressed PLHIV, whereby we imputed a value of 1 c/ml for PLHIV with undetectable VL. A country’s total VL comprising on unsuppressed VL values would not differ substantially from the values we show.

On page 12, I don't see HIV incidence data. Kindly clarify.
Response: Table 2 displays HIV incidence estimates.

On page 14, the statement "Except for CD4 T cell counts below..." may not true because the confidence intervals overlap. Also presenting the confidence intervals as 25% and 75% is not easy to follow, especially as these demarcations are not discussed in the discussion section. Please revert to the 95% CI or 90% CI, if not able to discuss in details the 25% and 75%.
Response: Thank you for this observation; we altered the original text (*Except for CD4+ T cell counts below 100/mm3, male ARV-negative PLHIV tend to exhibit higher VL than females*.) to say: “*Fig 2 displays the log10 VL by CD4+ T cell count category and sex; male ARV-negative PLHIV tended to exhibit higher VL than females*.”

Page 20, Table 3, analyzing correlation between population viremia and HIV incidence is not correct. HIV incidence is in population viremia. So you are measuring the same thing. If you want to go further with this metric maybe use another method such as instrumental variables. Maybe best to remove this table.
Response: A very small number of unsuppressed PLHIV in our survey samples were HIV-recent and as such (per our recent infection algorithm) have unsuppressed VL. These persons, like any person with unsuppressed VL, is at risk of transmitting HIV, hence this small subgroup is included in population viremia.

On page 22, second sentence regarding the PHIA sampling PLHIV should be moved to methods section. Similarly, the sentence after source 30, should be moved to the methods section.
Response: As suggested, we moved these two sentences to the Methods section.

Page 24 and 26. Cameroun and Côte D'Ivoire's sample size are generally smaller. Analysis focusing on these countries need additional investigations.
Response: Eswatini, Lesotho, and Namibia have smaller sample sizes than Cameroon and Cote d’Ivoire; perhaps you are referring to the number of PLHIV in these samples? It is not clear to us what additional investigations are referred to?

Page 25, first paragraph is very important and some effort should be spent on discussing this finding.
Response: Thank you, we agree that this is an important finding. At the end of this para, we added the sentence “These data also suggest that estimating the number of viremic PLHIV across demographic and behavioral strata has its own merit, beyond VLS monitoring.”

This is an important topic which with further revisions may add a lot to the body of knowledge on viral load metrics. I am happy to review further the revised manuscript.

Response: We thank you for the review and helpful comments made.

Reviewer #3: Reviewer Report

The epidemiology of HIV population viral load in twelve sub-Saharan African countries

The topic is relevant and important metrics were highlighted. The analysis are intense and well executed. However, was looking forward to seeing data from country like South Africa, which is the epicentre of HIV in sub-Saharan Africa. Not sure why the authors did not include it.
Response: South Africa’s SABSSM survey followed its own design, somewhat different from the PHIA, and the different structure of the data set prompted us to not use this survey’s data.

Introduction
Line 70 -71: The reference provided was for the 90-90-90 target, the revised 95-95-95 target needs to be referenced
Response: We edited the sentence slightly and altered the reference as suggested.

Material and methods
Line 97: Firstly, I suggest the subheading to read “Data Sources, Setting and Study design” instead of Setting and study design. Secondly the sub section did not clearly show the source of data but more of the study sponsors. Thirdly, a few more description of the study design is needful.
Response: We altered the subheading as suggested and added a sentence and reference with the location of the (downloadable) PHIA data sets. More detail about data collection and biomarker procedures are shown in the subsequent *Methods* sub-sections (in addition to the references shown).

Line 99: Grammar error. response efforts in “select low- and middle-income countries”, … Should be …in selected low- and middle-income countries …….
Response: Corrected.

Line 101: Grammar error. Implementing partners included country governments, grammar error …. should be …. “Implementing partners includes country governments
Response: We believe our sentence is grammatically correct (past tense).

Line 112: “The proportion of survey participants who consented to a blood draw” …… Relook at the sentence , need to be rephrased
Response: We rephrased the sentence to read “Among participants enrolled in the surveys the proportion who consented to a blood draw ranged from 87.5% to 99.7% (median: 92.5%).” Of note, individuals first consented to enroll in the survey overall, and then separately were asked to consent to a blood draw.

Line 108 -111: Shows various data used and number. Why was South Africa data not included. Was it intentional or was it not available.
However, from the introduction line 90-91, it was stated that data are becoming increasingly available with South Africa in reference. It will be good to include South Africa data in the analysis otherwise, the authors can remove South Africa from Line 91. As not having data from that country in the analysis make the statement contradicting.
Response: We deleted the reference to South Africa. We added a reference to Kenya instead.

Including South Africa data in the analysis is highly recommended. Good to see the correlation of the VL metrics from other countries and South Africa, especially Eswatini
Response: South Africa’s survey is not a PHIA and its design (and data structure) was slightly different from the 12 PHIAs in our analysis.

Line 113: The gender inclusion should be reflected in the participants.
Response: Added as suggested.

Line 222. …….by including all adults …. kindly state the age brackets for the adults.
Response: We replaced adults with participants – emphasis here is not on age but HIV status as we initially computed the Gini coefficients for PLHIV only and then recomputed the coefficients for all (HIV-pos and HIV-neg) adults. The country-specific age ranges are shown in Table 1.

Line 226: References or the ethical approval numbers from each country should follow the statement in this line.
Response: We include all IRB relevant information as required, thank you. Since more than a dozen IRBs reviewed all these PHIAs we will discuss with the journal how best to incorporate this information.

Data management and analysis
Line 196 to 199: The Gini coefficient and Lorenz curves were defined/ explained. Kindly Provide Reference(s). Furthermore. A brief explanation of the correlation range is needful. When do we say the correlation is strong, weak, or moderate (show by values)
Response: Reference numbers for Gini coefficients and Lorenz curves are shown at the beginning of this paragraph. We also state that “Gini coefficients are a measure of statistical dispersion, ranging from 0 (perfect parity) to 1 (perfect disparity), whereas Lorenz curves graphically depict disparity.” We refrained from qualifying Gini coefficients also because here (unlike with, say, income) inequality is a highly desired state.

Results
Line 235: Check grammar …. Table 1 shows select HIV and VL- related metrics. This should be ……Table 1 shows selected HIV and ……….
Response: Corrected

There was NO NUMBER LINE after 246 in the Results and Discussion sections. Thus, referring to any review was impossible. Adding number line to these sections is then recommended to enable further review.
Response: Apologies for this omission.

Tables and Figures looks good

Supplementary material
Figures in the Supplementary material should be relabelled as Figure S1, S2, ,,,,,, for clear distinction from the main document figures

Response: Sorry for any confusion; there are no supplementary figures. All figures are meant to be embedded in the manuscript’s main body.

6. PLOS authors have the option to publish the peer review history of their article ([what does this mean?](https://journals.plos.org/plosone/s/editorial-and-peer-review-process#loc-peer-review-history)). If published, this will include your full peer review and any attached files.


If you choose “no”, your identity will remain anonymous but your review may still be made public.


**Do you want your identity to be public for this peer review?** For information about this choice, including consent withdrawal, please see our [Privacy Policy](https://www.plos.org/privacy-policy).

Reviewer #1: No

Reviewer #2: **Yes:** David Chipanta

Reviewer #3: **Yes:** Adenike .O. Soogun

[NOTE: If reviewer comments were submitted as an attachment file, they will be attached to this email and accessible via the submission site. Please log into your account, locate the manuscript record, and check for the action link "View Attachments". If this link does not appear, there are no attachment files.]

While revising your submission, please upload your figure files to the Preflight Analysis and Conversion Engine (PACE) digital diagnostic tool, <https://pacev2.apexcovantage.com/>. PACE helps ensure that figures meet PLOS requirements. To use PACE, you must first register as a user. Registration is free. Then, login and navigate to the UPLOAD tab, where you will find detailed instructions on how to use the tool. If you encounter any issues or have any questions when using PACE, please email PLOS at [figures@plos.org](mailto:figures@plos.org). Please note that Supporting Information files do not need this step.
