# Supplementary material for: The epidemiology of HIV population viral load in twelve sub-Saharan African countries
Source: PLoS One. 2023 Jun 26;18(6):e0275560. doi: 10.1371/journal.pone.0275560 (PMC10292693; doi:10.1371/journal.pone.0275560)
Supplement: Attachment — Submitted filename: Rebuttal Letter - PONE-D-22-26006 - March 2023.docx [file pone.0275560.s008.docx]

Rebuttal letter

PONE-D-22-26006R1

The epidemiology of HIV population viral load in twelve sub-Saharan African countries

PLOS ONE

We very much thank the reviewer(s) for the renewed attention and remaining comments. Please find below in purple font our point-to-point responses to all larger comments made in the manuscript’s pdf version.

Please note: In response to a reviewer comment, we generated two new supplemental tables showing measures of uncertainty for the indicators displayed in Tables 1 and 2.

Reviewer #1: Most of my queries have been addressed by the authors. All the scientific questions have been addressed appropriately. The answer provided to my major comment is not satisfactory from an ethical point of view.

Reviewer #2: Many thanks for this useful paper and the hard work that has gone into it. The paper needs some minor revision in order for it to be ready for publication. Several issues need to be resolved: first the correlation between population viremia and HIV incidence is problematic because of the potential for reverse causality between population viremia and HIV incidence. Some of the discussions are tangental and can be taken out without losing much. I like that you recognise that the ratios are important but by themselves do not say much and need additional information to make them useful. The estimates need confidence intervals. I am sure you have this information.

Some of the information on the first page relating to the UNAIDS strategy and others are not correct. Please see how to rephrase.

Reviewer #3: Recommended for publication

**Abstract**

Comment: add the countries.i.e. from xxx in country y to x2 in Country y2

Response: Added.

Comment: replace female with women, through out the manuscript

Comment: please replace male with men

Response: The surveys providing the data for this manuscript probed participants’ sex at birth (male, female), not gender (woman, man). We do not know the gender of the survey participants. Also, when examining viral load, the more appropriate comparison is that between male and female as biologic males on average experience higher viral load. Hence, we suggest keeping to the term *male/female*.

Comment: because viral load and HIV infections are related, you have use a different methods such as instruments to measure the relationship between the two (NB: similar comment on Table 3)

Response: Out understanding is that instrument variable analysis generally is conducted at the individual level, not the aggregate level which our analysis uses. As well, the fraction of unsuppressed PLHIV that is due to incident HIV is small, and unsuppressed PLHIV with incident HIV are at risk of transmitting HIV just as unsuppressed PLHIV with prevalent HIV infection are. Further, we do not claim a causal inference between unsuppressed HIV and incidence but restrict ourselves to reporting measures of correlation. We also like to point to published literature that similarly examined the correlation between viral load metrics and HIV incidence without using IVA, such as:

Solomon, Sunil Suhas, et al. "Community viral load, antiretroviral therapy coverage, and HIV incidence in India: a cross-sectional, comparative study." *The Lancet HIV* 3.4 (2016): e183-e190.

Tanser, Frank, et al. "Effect of population viral load on prospective HIV incidence in a hyperendemic rural African community." *Science translational medicine* 9.420 (2017): eaam8012.

Comment: Unsurpressed is viremic. Maybe use one of these two terms consistently

Response: Thank you. We replaced viremic with unsuppressed.

**Introduction**

Comment: replace with an estimated 38 million people were living with HIV in 2021. The current wording means the burden of HIV covered 38 million people when you mean the burden of HIV was

Response: Changed as suggested

Comment - Line 67: Treatment is key part of the UNAIDS strategy, however it is not correct to say the strategy focuses on treatment as prevention. Please rephrase with wording from the strategy

Response: We edited and partially deleted this statement: “*In the absence of a vaccine or cure, Treatment as Prevention, a concept informed by clinical trials demonstrated that ART-mediated viral load suppression (VLS) minimizes the risk of onward HIV transmission[**2**].*”

Comment – Line 108: Please include ethiopia or indicate why you excluded the ethiopia data set in the analysis

Response: We added this text: “*We excluded the Ethiopia PHIA as sampling was restricted to urban areas and so did not allow for the examination of national VL metrics or the relationship between viremia and HIV incidence.*”

Comment – Line 114: including confirmatory laboratory test according to national testing algorithm

Response: Further below (Section “Biologic measures”) we state: “Venous blood collected with EDTA as an anticoagulant was tested at the household for HIV using the countries’ national rapid test algorithm. All HIV-positive specimens underwent confirmatory testing using the Geenius HIV 1/2 Supplemental Assay (Bio-Rad, Hercules, CA, USA) or PCR testing. Additional HIV quality control testing varied by country.”

Comment – Line 223: Then add, that for those infected with suppressed o copies we ordered as 2 or more? or by the absolute value of their viral load?

Response: We already state further up (Line 205): “Undetectable VL values were assigned a VL value of 1 for the construction of the Lorenz curves.”

Comment – Line 240: add the values such rangin from xx for men and x2 for women to xxx for me and xxx3 for women

Response: We altered the sentence (line 249+) to say: “In all countries examined, the mean log_10_ VL among viremic males (range: 4.47-5.15) exceeded that among viremic females (range: 4.25-4.75) and peaked for both sexes in Cameroon.”

Comment on Table 1 (and similar comments elsewhere): Since these are estimates, you should include 95% confidence intervals

Response: We prepared supplemental files with tables showing uncertainty ranges for most indicators displayed in the main manuscript’s tables.

Comment – Table 3: This is difficult to understand that there is a relationship between people who are HIV negative and HIV incidence

Response: While perhaps a less common metric, restricting the denominator to HIV-negatives reflects the notion that all incidence arises from the pool of HIV negatives.

Comment – Line 383 (revised manuscript), face a hyperendemic epidemic (i.e., <15% viremia prevalence among adults): put a source for these benchmarks

Response: Reference added: UNAIDS Terminology Guidelines. Geneva, October 2011. Accessed 21 Mar 2023 at <https://unaids-test.unaids.org/sites/default/files/unaids/contentassets/documents/unaidspublication/2011/JC2118_terminology-guidelines_en.pdf>.

Comment – Line 390: ….where testing, treatment, and successful VLS is achieved at a larger community level.: You did not test this in your study so you cannot make this inference

Response: We altered the sentence to say: “In other countries (Kenya, Cameroon, Namibia) clear differences emerged, apparent HIV “hotspots” subsided and “viremia hotspots” surfaced as distinct from HIV prevalence hotspots, illustrating areas with poor viral suppression.”

Comment – Line 432 (Lorenz curves illustrate the large-scale successes of most national ART programs that resulted in VLS in many or most PLHIV): You cannot make this claim, since the 1% or 5% might actually be people living with HIV who stopped treatment

Response: Whether the 1% or 5% with the highest VL represent ART-naïve PLHIV or PLHIV off ART, this does not influence the shape of the Lorenz curves which show the massive impact of ART on VLS by displaying the extreme and desirable ‘inequality’ of VL distribution.

Comment – Line 460 (…10 to 11 HIV transmission events per 100 unsuppressed adults per year): You did not measure HIV transmission events in your study. Where does this come from?

Response: We estimated HIV transmission ratios as originally described in Methods: “For each country we estimated the annualized HIV transmission ratios using the following formula: estimated number of annual new HIV-1 infections/number PLHIV * 100, thus deriving estimates of the number of HIV transmissions per 100 PLHIV per year. Assuming that all transmissions stem from unsuppressed PLHIV we further estimated *effective* HIV transmission ratios using the same formula but restricting the number of PLHIV to unsuppressed only. Assuming that all transmission was between opposite sexes we computed sex-specific effective HIV transmission ratios (male-to-female and female-to-male). Using the same data, we also estimated the number of unsuppressed adults per incident HIV infection for each country by dividing the number of unsuppressed adults by the number of incident HIV infections, for both sexes combined as well as for each sex (No. unsuppressed males / No. female incident HIV; No. unsuppressed females / No. male incident HIV), thus deriving a measure of how many unsuppressed adults/males/females on average lead to one incident case of adult/female/male HIV per year.”

Comment (Line 465: Cote d’Ivoire offered the lowest HIV transmission ratios, suggesting that unsuppressed adults on average pose comparatively little risk of onward transmission.): You cannot make this conclusion, because even with lower viremic ratios, behavorial issues offset the lower transmission ratios

Response: You are correct that behavioral traits may substantially alter the actual rate of transmission. However, as we say in this statement, Cote d’Ivoire showed the lowest estimated transmission ratios compared to other countries; this is simply a statement of comparison among examined countries.

Comment (Line 474ff, entire paragraph): Since you did not study transmisson in this study maybe take this out because it is tangental

Response: Please see our response further up related to this topic; we did estimate transmission ratios.

Comment (Line 506 - …can be estimated with more precision than HIV incidence in the survey or surveillance setting): Maybe take out this because it is difficult to estimate with precision for example that someone with viremia of 1001 copies has less transmission potention than someone with 1010

Response: While we appreciate this comment about the somewhat arbitrary nature of establishing a VLS threshold of 1000 copies/ml, this threshold is common in HIV focused surveys. The intent of our statement is to highlight the much greater feasibility for estimating VL metrics with satisfying precision compared to HIV incidence. For surveillance, this is an important benefit/advantage.
