# Supplementary material for: The epidemiology of HIV population viral load in twelve sub-Saharan African countries
Source: PLoS One. 2023 Jun 26;18(6):e0275560. doi: 10.1371/journal.pone.0275560 (PMC10292693; doi:10.1371/journal.pone.0275560)
Supplement: Attachment — Submitted filename: Rebuttal Letter - PONE-D-22-26006 - May 2023.docx [file pone.0275560.s010.docx]

Rebuttal letter, June 3, 2023

PONE-D-22-26006R1

The epidemiology of HIV population viral load in twelve sub-Saharan African countries

PLOS ONE

We very much thank the reviewer for the renewed review of our comments and manuscript revision. Please find below in purple font our point-to-point responses.

Comment page 1: indicate that it is available with a request to ICAP /Columbia PHIA (put the details of the website to which readers can get the data) as below. Just copy the site and paste it here.

Response: This response is the only applicable response option (drop down menu at PONE). We added this detail to the next text box that allows open ended text: “*Information about how to access and download PHIA data can be found here:* [*https://phia-data.icap.columbia.edu/*](https://phia-data.icap.columbia.edu/)*, including through this instructional video:* [*https://phia-data.icap.columbia.edu/about*](https://phia-data.icap.columbia.edu/about)”

Comment Line 63: , specifically East and Southern africa,

Response: Changed as suggested

Comment L63: sexual transmission of HIV

Response: Changed as suggested

Comment L69: by 2020,

Response: Changed as suggested

Comment L70: among PLHIV aware of their HIV positive status,

Response: Changed as suggested

Comment L71: reduces HIV-realted mortality and sexual transmission of HIV

Response: Changed as suggested

Comment L106: available to the public with a brief request to ICAP/Columbia, past the website

Response: We altered the sentence and referenced the website.

Comment L110: , negative, positive or undefined??

Response: Clarified (“unambiguous”)

Comment L122: Please see my comments about whether the analysis about Ethiopia should be included

Response: No changes were made to the text or analysis. As pointed out earlier, this was a study design decision as rural Ethiopia was not sampled. Also not included are data sets from other recently concluded PHIA. Other investigators may choose to analyze this data set or the data sets of other recently completed PHIA.

Comment: please write in full followed by an abbreviation if necessary (EDTA)

Response: Changed as suggested.

Comment L137+: You can replace this with a source.

Response: We altered the sentence and added a reference about VL-related laboratory procedures to say “*To determine VL, HIV-positive plasma specimens (or, if unavailable, dried blood spots (DBS), accounting for 3% of all participants) were further tested in-country using a Roche or Abbott platform.[**19**]*”

Comment L144: Since you are not covering CD4 analysis, maybe remove this information

Response: Thanks for this suggestion, but CD4 data are included in analysis as we show median VL by aggregate CD4 count.

Comment L151+: Maybe remove the names of company, city and country of the tests since you indicate that the details are published elswhere

Response: We removed company details as suggested.

Comment L245: add the 95% confidence intervals

Response: We added the 95% CI for Table 1 in a supplemental table: “*Table 1 shows selected HIV and VL-related metrics; corresponding measures of uncertainty are presented in Table S2.*”

Comment L246: Since these are estimates, you need to include the confidence intervals

Response: See comment above – 95% CI added through supplemental table.

Comment – Table 1 header: by country (Include 95% confidence intervals)

Response: See previous comments.

Comment L360-3: Please remove this because you don't know and also raises the question where the 300 and 35 comes from.

Response: Removed

Comment L363-7: same as above, maybe remove this sentence

Response: Removed

Comment (on previous rebuttal letter): I agree male/female is more precise than men or women. Men or women read considerably more humane, and does not lose its precision in this case. You can make a qualifying statement on the first use of male/female such as we refer to men as biogical male aged 15 years or older and women as biological females 15 years older unless indicated.

Response: We altered a statement in Methods to say “*Original data variables included household characteristics, demographics (including their sex at birth, hence the terms ‘male’ and ‘female’), HIV-related service uptake, and HIV-related risk behaviors.*”

Comment (on our comment in previous rebuttal letter): You can do instrumental variables at the aggregate level. There are other ways also to do something population viral load to break the potential reverse causality with HIV incidence. For example, Tanser, Frank, et al. "Effect of population viral load on prospective HIV incidence in a hyperendemic rural African community." Science translational medicine 9.420 (2017): eaam8012 had to do something to population viral load in order to use it in the HIV incidence measurement.

May use the method Tanser, Frank, et al. did to viral load and then see if it is correlated with incidence.

Solomon, Sunil Suhas, et al. "Community viral load, antiretroviral therapy coverage, and HIV incidence in India: a cross-sectional, comparative study." The Lancet HIV 3.4 (2016), e183-e190 proove my point that population viral load and HIV incidence may be one and the same. The correlation is so strong that you are measuring the same thing.

Response: We added to Discussion/limitations paragraph: “*Our surveys’ cross-sectional design does not allow us to infer a causal relationship between viremia and incidence; as well, all our surveys’ recent HIV infections were viremic as defined per our testing algorithm for recent HIV-1 infection.*”

Our manuscript does not claim a causal effect between unsuppressed HIV and transmission, numerous other studies have done that already. We added this statement to Table 3’s legend: “*R^2^ values should not be interpreted as a causal relationship between VL metrics and incident HIV*.”

Comment (in previous rebuttal letter): Even if it is restricted to urban areas you can do the analysis restricting it only to urban areas. Could you please include the analysis for Ethiopia? It will be a shame to exclude the analysis for Ethiopia just because it was conducted in urban areas

Response: Please also see our other response further above. While we appreciate the reviewers’ view on the added utility by including Ethiopia we are confident that our main observations would not change by including this country; instead the lack or rural Ethiopia in the sample would complicate analysis, display, and interpretation of several indicators.

Comment (to comment in previous rebuttal letter: Indicate the mean point estimate for men here AND:

Indicate the mean point estimate for women here. Is the range the 95% confidence interval? It looks like the ranges overlap, showing that the unsuppression of men do not strictly exceed that of women.

Response: This comment refers to manuscript text shown at Line 251ff. We altered the text to say “*In each country examined, the mean log_10_ VL among unsuppressed males (range across countries: 4.47-5.15) exceeded that among unsuppressed females (range across countries: 4.25-4.75); however, 95% CI for some countries overlapped (Table S2). The highest mean log_10_ VL for both males and females were observed in Cameroon.*” Please note that the median unsuppressed log10 VL for males (4.73) and females (4.49) (and for both sexes combined) is shown in Table 1 (last column).

Comment (on comment in previous rebuttal letter): Use the latest version: https://www.unaids.org/sites/default/files/media_asset/2015_terminology_guidelines_en.pdf

Response: Thank you for pointing out this newer reference. We updated it.

Comment (on comment in previous rebuttal letter): You suggestion is problematic: "...unsuppressed adults on average pose comparatively little risk of onward transmission."

In fact this may not be correct. It is unsurpressed adults who pose risk! Coud you please modify this?

Response: To quote the full sentence: “*Cote d’Ivoire offered the lowest HIV transmission ratios, suggesting that unsuppressed adults on average pose comparatively little risk of onward transmission.*” In an attempt to make our statement clearer we altered this sentence to say “*Cote d’Ivoire offered the lowest estimated HIV transmission ratios, whereas Cameroon saw the largest difference between male-to-female and female-to-male transmission ratios*.”

PS: Please note that we changed the chart type for Figure 2.
